# Different Paths, Same Structure: “Developmental Systems Drift” at Work

**DOI:** 10.1371/journal.pbio.1001113

**Published:** 2011-07-26

**Authors:** Richard Robinson

**Affiliations:** Freelance Science Writer, Sherborn, Massachusetts, United States of America

**Figure:**
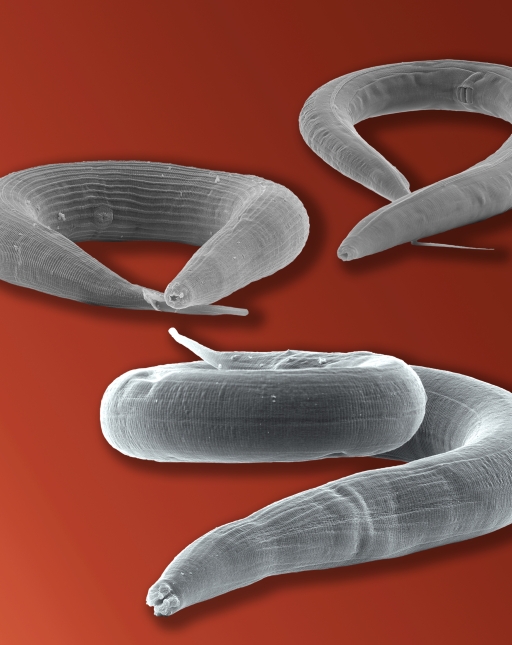
A diversity of molecular mechanisms modified vulva induction in the free-living nematodes *Pristionchus pacificus* and *Caenorhabditis elegans*.

Closely related species often bear many similarities in outward appearance—think of the elongated snout of wolf and dog, or the opposing twigs on Norway and sugar maples. The parsimonious explanation for similar features is that they arise by similar developmental mechanisms, but an emerging concept in evolutionary development suggests this may not always be so. “Developmental systems drift” proposes that so long as the morphological feature itself doesn't change (thus preserving the organism's interaction with its environment), the underlying molecular pathway specifying it can be altered without evolutionary penalty.

While it is an intriguing hypothesis, there have been few studies that offer evidence for its operation in nature, not surprisingly, given the degree of molecular detail needed to confirm or refute it. In this issue of *PLoS Biology*, Xiaoyue Wang and Ralf Sommer elucidate those details to show that in two species of roundworm, similar sexual structures arise from marked differences in signaling pathways, differences involving both novel wiring and novel protein modifications.

*Caenorhabditis elegans* and *Pristionchus pacificus* are members of separate roundworm families whose last common ancestor lived at least 250 million years ago. Nonetheless, they share many features, including a vulva that arises from the same set of precursor cells. In *C. elegans*, the induction of the vulvar precursor cells is under the control of a signaling pathway employing epidermal growth factor and RAS, while in *P. pacificus*, induction relies on Wnt signaling. Furthermore, in *P. pacificus*, signaling emanates in part from the posterior body region, and involves a unique ligand-receptor interaction; neither of these features is found in *C. elegans*.

To understand the details of vulvar induction in *P. pacificus*, the authors explored the molecular effects of three genes: *Ppa-lin-17*, *Ppa-lin-18*, and *Ppa-egl-20*. Both *lin-17* and *lin-18* are membrane receptors, and bind *egl-20*. Binding of *egl-20* to *lin-18* induces vulvar formation. Not so for *lin-17*, however. Either suppressing *lin-17*, or overexpressing *egl-20*, leads to vulvar induction. This suggested to the authors that *lin-17*'s role in vulvar induction is inhibitory, and its binding of *egl-20* serves as a brake on *lin-18*/*egl-20* signaling.

Additional support for this model came when they found that both *lin-17* and *egl-20* were expressed in the posterior body region, consistent with the source of vulvar induction signaling in *P. pacificus*.

Further experiments shed light on other details of the signaling system. The authors found a *lin-17* mutation that drove ectopic multiple vulva formation. But this mutation was not a null allele, and its mechanism differed from that of the mull mutant. Instead, the mutation caused a 17-amino acid domain to be added to the intracellular end of the protein, and this extension served as an SH3 binding motif, a motif known as a common binding domain. Curiously, they found the same domain on *lin-18*.

The authors showed that the job of the domain on *lin-18* was most likely to bind an inhibitor to prevent Wnt signaling and vulva induction in the absence of *egl-20*. When the domain was expressed on *lin-17*, they speculate, it depleted the inhibitor from its normal position on *lin-18*, leading to excess Wnt signaling and ectopic vulva formation.

Finally, they showed that the normal signaling system requires the downstream participation of two proteins, Axin and a beta-catenin, both known members of other Wnt signaling pathways.

None of the above is found in *C. elegans*, the authors note, indicating the degree of developmental dissimilarity between the two species in accomplishing the same morphological result—the very definition of developmental systems drift. They also point out that the central role of the SH3 binding motif uncovered in this study suggests the potential role for evolution of small peptides in causing major developmental changes.

**Wang X, Sommer RJ (2011) Antagonism of LIN-17/Frizzled and LIN-18/Ryk in Nematode Vulva Induction Reveals Evolutionary Alterations in Core Developmental Pathways. doi:10.1371/journal.pbio.1001110**

